# *N*-Butyl-2-cyanoacrylate-based injectable and *in situ*-forming implants for efficient intratumoral chemotherapy

**DOI:** 10.1080/10717544.2017.1309478

**Published:** 2017-04-25

**Authors:** Yanpu Wu, Luming Wang, Kaili Zhang, Lixiao Zhou, Xiaobing Zhang, Xuecheng Jiang, Chenggang Zhu

**Affiliations:** 1 College of Life Sciences, Zhejiang University, Hangzhou, Zhejiang, China and; 2 Jiaxing Maternity and Child Health Care Hospital, Jiaxing, Zhejiang, China

**Keywords:** NBCA, *in situ*-forming implants, paclitaxel, epirubicin, intratumoral chemotherapy

## Abstract

The local delivery of chemotherapeutic drugs to tumor sites is an effective approach for achieving therapeutic drug concentrations in solid tumors. Injectable implants with the ability to form *in situ* represent one of the most promising technologies for intratumoral chemotherapy. However, many issues must be resolved before these implants can be applied in clinical practice. Herein, we report a novel injectable *in situ*-forming implant system composed of *n*-butyl-2-cyanoacrylate (NBCA) and ethyl oleate, and the sol–gel phase transition is activated by anions in body fluids or blood. This newly developed injectable NBCA ethyl oleate implant (INEI) is biodegradable, biocompatible, and non-toxic. INEI solidifies in several seconds after exposure to body fluids or blood, and the implant’s *in vivo* degradation time can be controlled. In addition, the pore sizes formed by the polymerization of NBCA can be decreased by increasing the NBCA concentration in the implants. Therefore, the drug retention/release time can be adjusted from a few weeks to several months by changing the concentration of NBCA in the implant formulation. Anti-tumor experiments in animal models showed that the average growth inhibition rate of xenografted human breast cancer cells by the paclitaxel-loaded INEI (40% NBCA) was 80%, and they also indicated that tumors in some of the mice were completely eliminated by just a single dosage injection. For the epirubicin-loaded INEI (50% NBCA), the average growth inhibition rate of xenografted human liver cancer cells was 58%. Thus, the chemotherapeutic drug-loaded INEIs exhibited excellent therapeutic efficacy for local chemotherapy.

## Introduction

Cancer is a serious disease that poses a threat to human life. Currently, surgical resection followed by adjuvant chemotherapy or radiation therapy is the most curative treatment option for solid tumors (Maluccio & Covey, 2012; Dieli-Conwright et al., 2014). Because chemotherapy drugs do not present specificity for cancer cells, only a small fraction of the total dose reaches the tumor site. Thus, only a low concentration of the drug reaches the tumor site, which reduces the efficacy of the tumor treatment. In addition, the remainder of the dose can cause serious side effects on healthy organs and tissues because of the systemic distribution of the drug via the blood (Jain, 1998). Therefore, physical or chemical methods of enhancing anti-tumor activity by increasing the targeting ability and lowering the side effects of these drugs have been employed.

Injected intratumoral drugs have long been used in the treatment of tumors (Kanekal et al., 2009; Xie et al., 2012). Studies have explored injecting ethanol into a solid tumor to achieve ablation, and this course of treatment results in a certain therapeutic effect (Giorgio, 2010). Other attempts to improve the treatment of tumors have focused on injecting chemotherapeutic drugs into tumors via image guidance, which has been successfully implemented in the treatment of tumors in the brain (Duvillard et al., 2007), lung, pancreas, and liver (Chakravarty et al., 2015). In addition, transcatheter arterial chemoembolization (TACE) for liver tumor treatment has been successful and can increase the concentration of chemotherapy drugs within the tumor by 10–100 times while reducing systemic toxicity (Guan et al., 2012; Han et al., 2014). Commonly used embolization agents include iodine oil and gelatin (Llovet & Bruix, 2003; Yamashita et al., 2009). However, the main drawback of intratumoral injections of drug solutions or TACE is the rapid clearance of the drugs, which precludes the tumors from being exposed to the drugs for the necessary amount of time (El-Kareh & Secomb, 2000; Guan et al., 2012).

Therefore, researchers have developed intratumoral drug-releasing implants for tumor treatment that have shown increasing promise in recent years (Sathornsumetee & Rich, 2006; Lee et al., 2010). These implants can be divided into two categories. One category is the solid implant, which is an implantable device composed of polymers that contain either radioactive elements or chemotherapeutic drugs. These implants have a drug release time of up to a few weeks or months, and they have been approved for the clinical treatment of prostate and brain cancers. However, solid implants also have disadvantages: implants require a small incision near the tumor or a special syringe to inject the device into the patient. If the material degradation is not good, then inflammation is typically observed, and surgery will be required to remove the device. More importantly, these solid implants are difficult to implant in the body's internal organs.

The second type of implant is an injectable *in situ*-forming implant that is injected into the body under physiological conditions and converted into a solid or semi-solid drug depot, which is typically polymeric (Weinberg et al., 2008; Madan et al., 2009). These implants can be divided into several types depending on their formation mechanism: thermoplastic pastes, *in situ* crosslinked systems (Huynh et al., 2012; Lu et al., 2014), thermally induced sol–gel transition systems (Jeong et al., 2002; Yang & El Haj, 2006), *in situ*-solidifying organogels (Solano et al., 2013), etc. The precipitation of these types of *in situ*-forming implants can be triggered by pH (Namkung & Chu, 2007), temperature (Jeong et al., 2002; Lin et al., 2014), or solvent exchanges (Casuso et al., 2015). This strategy has several advantages, including the ability to inject these depots as a liquid through a needle or a catheter without surgical assistance (Li et al., 2012), which allows for the easier administration of drugs at multiple sites. However, these types of *in situ*-forming implants also have some disadvantages. For example, the thermoplastic paste systems must be heated and softened before use, and the high temperature required for softening will cause pain at the site of the injection. The thermally induced sol–gel transition systems have a high water content, which generates an obvious hydrophilic drug burst release phenomenon. *In situ*-solidifying organogels are influenced by many factors and have a complex preparation process.

**Figure 1.**
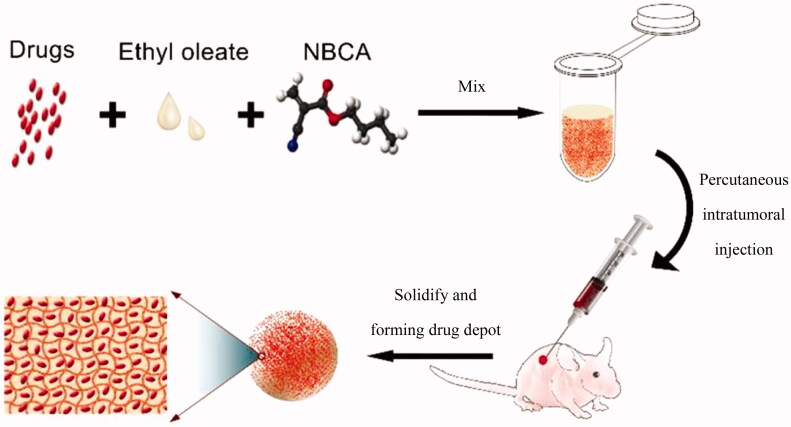
Schematic of the injectable and *in situ*-forming implants for tumor therapy. This injectable implant presents a liquid–solid phase transformation that is responsive to body fluid or blood. Chemotherapy drugs and ethyl oleate can be prepared in advance and stored in the refrigerator for a long time. Before use, the drug and ethyl oleate suspension are mixed with the NBCA and then injected into the animal body. NBCA will polymerize immediately at the injection site, thus forming a drug depot.

Herein, we report the first delivery system that is based on *n*-butyl-2-cyanoacrylate adhesive (NBCA) and ethyl oleate. This system can be used as an injectable implant for efficient local chemotherapy, with the sol to gel phase transition triggered by anions in body fluids or blood, such as the hydroxide anion. The schematic of the injectable and *in situ*-forming implants for tumor therapy is shown as Figure 1. Compared with the other type of injectable *in situ*-forming implants, the NBCA-ethyl oleate implant (INEI) is more flexible, more convenient, and more efficient for intratumoral chemotherapy in clinical applications.

## Materials and methods

### Materials

*N*-Butyl-2-cyanoacrylate was purchased from Jinpeng Chemical Co., LTD, Zhejiang, China. Paclitaxel and epirubicin were purchased from Haizheng Pharmaceutical Co., LTD, Zhejiang, China. Taxol was purchased from Shiqiao Biological Pharmaceutical Co., LTD, Beijing, China. Ethyl oleate was purchased from Aladdin Chemicals Co., LTD, Shanghai, China. The fluorescent probe 1,1-dioctadecyltetramethyl indotricarbocyanine iodide (DiR) was purchased from Biotium, Fremont, CA. Dialysis tubes (molecular weight cutoff 12 000–14 000 Da) were purchased from Spectrum Co., LTD., Fort Worth, TX. Human breast cancer cell line MDA-MB-231 was obtained from the cell bank of the Chinese Academy of Sciences, Shanghai, China. Female nude mice (4 weeks old) and ICR mice were purchased from SLAC Laboratory Animal Co. LTD, Shanghai, China and maintained under SPF conditions for 1 week before the study and provided access to standard food and water. All of the other reagents and solvents were purchased from Sinopharm Chemical Reagent Co., LTD., Mainland, China, and used as received. All the studies comply with the principles of care and use of laboratory animals from the Institutional Animal Care and Use Committee of Zhejiang University Health Science Center.

### Preparation of the drug–ethyl oleate suspension

Paclitaxel was dissolved in ethanol to a final concentration of 60 mg/mL and then added to a solution of ethyl oleate with a volume ratio of 1:3, and the solution was stirred with a magnetic stirrer for 30 min. The solvent was removed from the resulting mixture using a freeze-dry system for 4 h. Then, the paclitaxel-ethyl oleate suspension was sonicated three times using a probe-type sonicator at 200 W for 2 min in an ice bath. The pulse was turned off for 2 s every 4 s to prevent an increase in temperature. Finally, the paclitaxel-ethyl oleate suspension (20 mg/mL) was stored at 4 °C for subsequent use. Epirubicin powder was directly added into the ethyl oleate solution and then sonicated using the process mentioned above. The fluorescent probe DiR was dissolved into ethanol at a concentration of 1 mg/mL. One microliter of the DiR-ethanol solution diluted in 1 mL ethyl oleate was obtained, and it yielded a 1 μg/mL DiR-ethyl oleate solution. All of the experiments were performed in the dark.

### Preparation of NBCA implants *in vitro*

For the implants prepared *in vitro*, 60 μL NBCA was added to a plate bottom tube with 140 μL a paclitaxel-ethyl oleate suspension or an epirubicin-ethyl oleate suspension, and the final NBCA concentration was 30%. Then, 2 μL saline was added, and the resulting solution was vortexed for 10 s and then left to sit at room temperature for 24 h.

### Electron microscope observations of the implants

The morphology of the paclitaxel particles in the implant was observed by transmission electron microscopy (TEM) (JEM1200EX, Japan Electron Optics Laboratory, Tokyo). Different concentrations of the NBCA implants were observed by a field emission scanning electron microscope (FESEM) (SU-8010, Japan Electron Optics Laboratory, Tokyo).

### *In vitro* drug release

Fifty microliters of INEI that contained either 30% NBCA and 0.1 mg paclitaxel or 50% NBCA and 0.1 mg epirubicin was added to a pre-swollen dialysis tube. Then, the dialysis tubes were immersed in a glass bottle with a sodium salicylate solution (100 mL for paclitaxel and 50 mL for epirubicin) (Huh et al., 2005). The clinical paclitaxel drug Taxol (Shiqiao Biological Pharmaceutical, Beijing, China) or epirubicin powder was added to the corresponding dialysis tubes as a control. The glass bottles were placed in a thermostatic shaker (speed at 40 rpm/min, 37 °C). At each time point (12 h, 1 d, 2 d, 3 d … 8 d), the solution around the dialysis tube was collected and replaced by an equal volume of the same medium. The paclitaxel solution (100 mL) of each group was extracted by a 25 mL methyl *tert*-butyl ether, and the extract was freeze dried and redissolved with 100 μL methanol. The concentration of paclitaxel dissolved in methanol was assayed by HPLC-UV using a C18 column with the following mobile phase: methanol–acetonitrile–water (4:3:3, v/v) at a flow rate of 1 mL/min and a detection wavelength of 228 nm, with a sample injection volume of 20 μL. The epirubicin solution was assayed directly by HPLC on a C18 column with the following mobile phase: acetonitrile-0.02 M NaH2PO4-triethylamine (34.0:66.0:0.3, v/v) adjusted to pH 4.0 with phosphoric acid. The detection was done using a fluorescence detector at *λ* ex 480 nm and λ em 550 nm, the flow rate was 1.0 mL/min, and the volume of injected sample was 20 μL.

### Biodegradability of NBCA implants

Thirty-two female ICR mice (7-weeks-old) were randomly divided into four groups, and 100 μL INEI mixtures with different volume ratios of NBCA (20%, 30%, 40%, and 50% NBCA) were injected into each mouse in the different groups by subcutaneous injection, with three parallel injection points on each mouse. One mouse from each group was sacrificed at 1 week intervals, and the implants were taken out and weighed.

### *In vivo* release of the fluorescent dye DiR

Nude mice were used as a tumor-bearing animal model. An MDA-MB-231 human breast cancer cell suspension (100 μL, containing 106 cells) was subcutaneously injected in the right armpit of each female nude mouse (5 weeks old). The tumors were measured twice a week by a digital caliper, and the experiment began when the tumors reached approximately 150 mm^3^. Ten nude mice bearing human MDA-MB-231 tumors were randomly divided into two groups. Each group received a single intratumoral injection of 50 μL of an INEI (30% NBCA)-DiR solution or 50 μL of a DiR-ethyl oleate solution. NIR imaging was performed after administration at 3 h, 3 d, 7 d, 11 d, 16 d, and 20 d by CRi Maestro *In Vivo* Imaging System (CRi, Woburn, MA) with a wavelength detection range of 750–800 nm. Prior to imaging, the mice were anesthetized with diethyl ether.

### *In vivo* anti-tumor activity

Thirty-five nude mice bearing human MDA-MB-231 tumors were prepared as described in the Methods section. When the tumors grew to approximately 120 mm^3^, the mice were randomly divided into five groups, with seven mice in each group. After the mice had been weighed and their tumors were measured, each group of mice was given a single intratumoral injection of 30 μL of either saline, INEI with 30% NBCA, Taxol (containing 0.3 mg paclitaxel), INEI with 0.3 mg paclitaxel and 30% NBCA, or INEI with 0.3 mg paclitaxel and 40% NBCA. The treatment efficacy was assessed by measuring the volume of the tumor with a caliper every 2 days. The tumor volume was calculated by the following formula: *V* = (length) × (width)2/2, where length (mm) and width (mm) are the tumor dimensions at the longest point and the widest point, respectively (Mamot et al., 2005). On day 20, all of the mice were sacrificed, and all of the tumors were weighed and ground up. The drug residue was then extracted and assayed by HPLC.

Using the same procedure, human HepG2 cells were utilized to establish the nude mice model of hepatocellular carcinoma. The HepG2 cell suspension (100 μL, containing 106 cells) was subcutaneously injected in the right armpit of each female nude mouse (5 weeks old). When the tumors grew to approximately 300 mm^3^, the HepG2 tumor-bearing nude mice were randomly divided into four groups, with five mice in each group. After the mice were weighed and their tumors were measured, each group of mice was administered a single intratumoral injection of 30 μL of either saline, INEI with 50% NBCA, epirubicin-PBS solution (containing 0.15 mg epirubicin), or INEI with 0.15 mg epirubicin and 50% NBCA. The following experiment was the same as for the paclitaxel treatment groups.

### Histological examination

To further evaluate the anti-tumor efficacy after performing the treatments with various formulations, the tumors were dissected from the mice and sectioned for a H&E assay for the pathology analysis.

### Statistical data analysis

Independent experiments were performed at least three times and triplicate samples were analyzed in each experiment. The values are expressed as the mean ± standard deviation (SD). A two-sample *t*-test was used to measure the statistical significance between pairs of results. A *p* value of < 0.05 was considered significant.

## Results

### Preparation of the drug–ethyl oleate suspension

**Figure 2.**
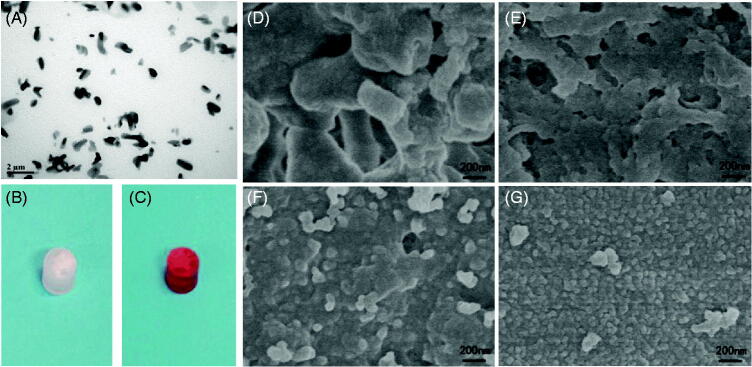
(A) TEM image of the paclitaxel-ethyl oleate suspension. The particles of paclitaxel in the ethyl oleate suspension are rice shaped, the length is approximately 0.5–1 μm, and the diameter is 0.5 μm. (B and C) Drug-loaded NBCA-ethyl oleate implants prepared *in vitro*. (B) Paclitaxel-loaded 30% NBCA of INEI; (C) epirubicin-loaded 30% NBCA of INEI. When water or deionized water was not added, the NBCA implants did not solidify for several days. Once a small amount of saline was added, the implants quickly solidified. (D–G) FESEM images of the NBCA-ethyl oleate implants. (B) 30% NBCA, (C) 50% NBCA, (D) 80% NBCA, and (E) 100% NBCA. NBCA is triggered by the anion and then self-crosslinks to form particles, which then cross-link with each other to form a solid material. The larger the particles, the greater the gaps formed between the particles.

Paclitaxel is a natural anticancer product because of its unique mechanism, and it has been applied to tumor therapy alone or in combination with other anticancer therapeutic agents. This drug has excellent therapeutic efficacy for a wide spectrum of cancers, especially for ovarian and breast cancers. Paclitaxel is a hydrophobic molecule and has poor solubility in water. To ensure that paclitaxel does not block the injection needle, the paclitaxel used in implants must be composed of tiny particles. To prepare the paclitaxel suspension, an ethanol solution of paclitaxel was added to ethyl oleate, and then the ethanol was removed by freeze drying. After ultrasonication, the paclitaxel-ethyl oleate suspension was slightly clearer and more fluid. TEM showed that paclitaxel in this suspension was rice shaped and presented lengths of approximately 0.5–1 μm and diameters of 0.5 μm (Figure 2A). The size and the shape of the paclitaxel particles in the suspension did not change after storage at 4 °C for 2 months.

### NBCA implant preparation and electron microscopy observations

Once the ratio of NBCA in the implant was higher than 20%, the NBCA–ethyl oleate mixture quickly solidified after adding a small amount of saline, and the hardness of the implant increased at higher NBCA ratios (Supplemental materials). Implants that contained 30% NBCA and paclitaxel or epirubicin cylinders were prepared *in vitro*, and these results indicated that the addition of drugs did not affect the curing of the implants and showed that the anions in the saline solution were sufficient to stimulate NBCA polymerization and promote the solidification of the implants (Figure 2B and C). We used FESEM to observe the NBCA–ethyl oleate implant. We found that with increases of the NBCA concentration, the irregular particles polymerized by NBCA become smaller and that the inner hole of the implant also became smaller. With 100% NBCA, the NBCA particle size was approximately 500 nm, and an obvious channel was no longer observed within the implant (Figure 2D–G).

### *In vitro* drug release

**Figure 3.**
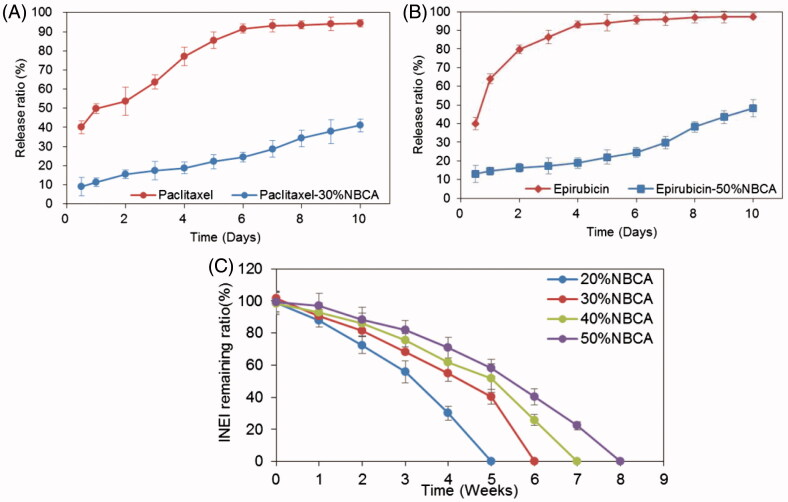
Time course of the drug release ratio *in vitro*. (A) Release ratio of paclitaxel and paclitaxel loaded in an INEI with 30% NBCA; and (B) release ratio of epirubicin and epirubicin loaded in an INEI with 50% NBCA. The concentration of released paclitaxel and epirubicin in the sodium salicylate solution was quantified as described in the Materials and methods section. The amount of initially incorporated molecules in the NBCA implants is defined as 100%. Each data point represents the mean ± SD of triplicate measurements. (C) Biodegradability of the NBCA implant *in vivo*. The implants were subcutaneously injected into the mice, removed after 1 h and weighed, and this weight was defined as 100%. One mouse from each group was sacrificed at 1 week intervals, and then the implants were removed and weighed. Each data point represents the mean ± SD of triplicate measurements (*n* = 3).

To evaluate the sustained release of drugs from the NBCA implants, the drug release experiments have been performed *in vitro*. To increase the solubility of paclitaxel in solution and accelerate the experimental process, we performed an *in vitro* release test in 1 mol/L pH 7.4 sodium salicylate solution at 37 °C under sink conditions. Figure 3(A) shows the release profiles of paclitaxel from the 30% NBCA implant and from Taxol only. The results demonstrated that the initial burst effect was lower (9.0% versus 40%) for the 30% NBCA implant versus Taxol. After 6 d, the total drug released from the 30% NBCA group was approximately 25%, whereas the total drug released from the Taxol group was 91.5%, which was close to a complete release of the drug. In addition, the total amount of drug released from the 30% NBCA group by the 10th day was 41%. Epirubicin is a more hydrophilic molecule; thus, a 50% NBCA implant and 20 mmol/L pH 7.4 PBS were used to conduct the *in vitro* release experiments. Figure 3(B) shows the release profiles of epirubicin from the 50% NBCA implant and from epirubicin powder. The data demonstrate that the initial burst effect was lower (13% versus 40%) for the epirubicin-loaded implants versus epirubicin powder. After 4 d, the total drug release from the 50% NBCA groups was approximately 19%, whereas the total drug release from the epirubicin powder group was 93%, which was close to a complete release of the drug. In addition, the total amount of drug released from the 50% NBCA group by the 10th day was 48.2%. These results indicate that the NBCA implants could significantly reduce the burst release effect and had a significant effect on the sustained release of both hydrophobic and hydrophilic drugs.

### Biodegradability of NBCA implants

The biodegradability of the implant material is a necessary feature for clinical applications but also has an important influence on the release of drugs from the implant. We injected different concentrations of NBCA subcutaneously into the mice and observed the degradation of the implants. The results showed that the 20% NBCA implant was completely degraded in 6 weeks and the 50% NBCA implant was completely degraded in 8 weeks. This result confirmed that the NBCA–ethyl oleate implants had good biodegradability *in vivo* and showed that increasing NBCA concentrations prolonged the degradation time of implants (Figure 3C). This characteristic increases the flexibility of this NBCA implant system for practical applications.

### *In vivo* release of the fluorescent dye DiR

DiR is a lipophilic, near-infrared fluorescent cyanine dye that is ideal for staining the cellular membrane. The maximum absorption wavelength of DiR is 748 nm, and its maximum emission wavelength is 780 nm. The near-infrared property of this dye makes it ideal for *in vivo* imaging because of the significantly reduced autofluorescence from animals at higher wavelengths. To directly observe the effect of the NBCA implants on the sustained release of drug molecules, one group of tumor-bearing mice received a single intratumoral dose of 50 μL DiR with the 30%.

NBCA implant and another group received an injection with 50 μL DiR and the ethyl oleate solution as a control. NIR imaging was performed at 3 h, 3 d, 7 d, 11 d, 16 d, and 20 d after administration.

**Figure 4.**
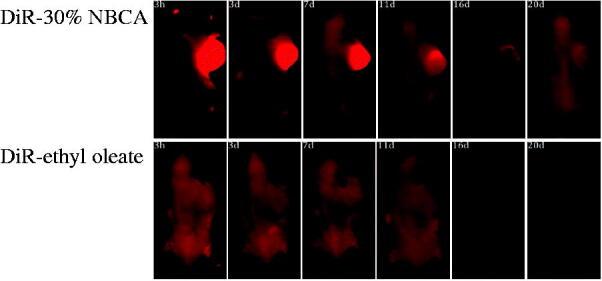
Effect of the NBCA implant on the retention of fluorescent dye DiR in the tumor: DiR-30% NBCA (top) and DiR-ethyl oleate solution (bottom).

The result shows that in the DiR-ethyl oleate group, fluorescence was observed throughout the entire body of the mice after 3 h and the fluorescence intensity of the tumor at the injection site was similar to that in other parts of the mice body (Figure 4). This observation indicated that the DiR dye had spread throughout the whole body and ethyl oleate had no effect on the sustained release of DiR because DiR did not remain near the injection site. However, bright DiR fluorescence was observed at the tumor site of the DiR-30% NBCA implant group, and over time, the fluorescence intensity at the tumor gradually decayed up to the 20 d. At this time point, the DiR fluorescence of the control group had completely disappeared. These results showed that the NBCA implant can retain the drug at the injection site, significantly retard the drug release to the entire body, and significantly affect the sustained release of the drug *in vivo*.

### Anti-tumor experiments

**Figure 5.**
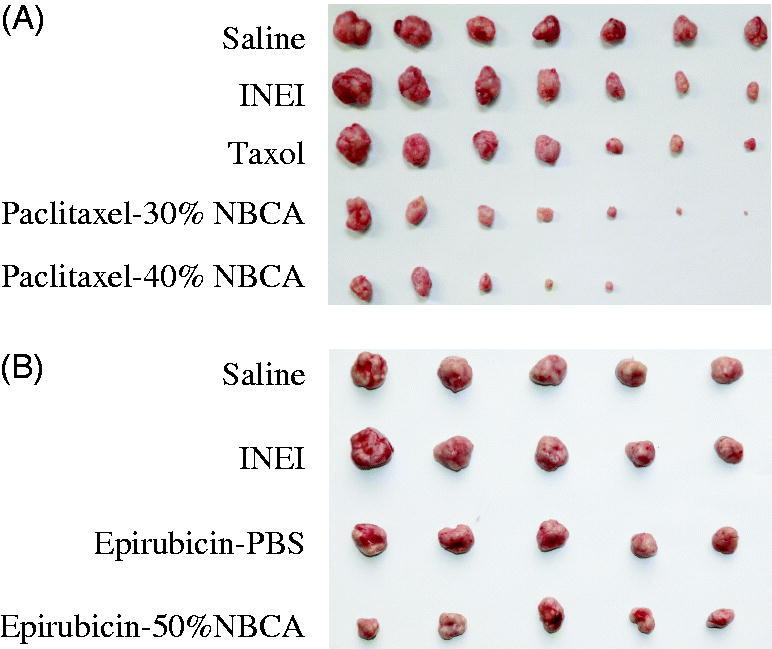
Drug-loaded INEI for tumor therapy *in vivo*. (A) Paclitaxel-loaded INEI for human xenograft breast tumor therapy (*n* = 7). (B) Epirubicin-loaded INEI for human xenograft liver tumor therapy (*n* = 5).

We evaluated the effect of the paclitaxel-loaded NBCA implant on the treatment of breast cancer using an animal model of human breast cancer. After 20 d of treatment, the mice were sacrificed and the tumors were removed. The average tumor weight from the saline group and the NBCA implant group (no paclitaxel) was approximately 1.40 g, indicating that the NBCA implant itself had no effect on the tumor growth (Figure 5A). In the Taxol group, the paclitaxel-loaded 30% NBCA implant group and paclitaxel-loaded 40% NBCA implant group, the average weight of the tumors was 0.79 g, 0.45 g, and 0.29 g, respectively, and the growth inhibition rate of the tumors was 44%, 68%, and 80%, respectively. For the animal model of liver cancer, the average tumor weight of the saline group and the NBCA implant group was 2.18 g and 2.14 g, respectively (Figure 5B). In the epirubicin-loaded PBS and epirubicin-loaded 50% NBCA implant groups, the average weight of the tumors was 1.58 g and 0.91 g, respectively, and the growth inhibition rate of the tumor was 28% and 58%, respectively.

These animal experiments show that NBCA implants alone do not alter the natural progression of the tumor, whereas the paclitaxel-loaded NBCA implants have a significant anti-tumor effect. In the 40% NBCA group, tumors in two mice completely disappeared. These results may be caused by the higher NBCA concentration, the higher NBCA cross-linking density, and the smaller pore size. The drugs may be released into the tumors more slowly, thereby increasing their drug exposure time. Compared with the direct intratumoral injection of the drug solution, the drugs released from the INEI had better tumor therapeutic effects. In a separate experiment, three groups of tumor-bearing mice were injected intratumorally with paclitaxel-loaded INEIs 1, 2, or 3 times. The results showed that the tumors of the single dosage group were significantly reduced, whereas three mice tumors disappeared from the double weekly injection dosage group (*n* = 8), and six tumors disappeared from the triple dosage group (*n* = 8). These results showed that increasing the number of injections (i.e. increasing the amount of INEIs) is likely to completely eliminate the tumor (Supplemental materials). In addition, the injection of INEIs not only increased the efficacy of hydrophobic drugs but also significantly increased the therapeutic effect of hydrophilic drugs on tumors.

After cutting open the tumor tissues at the end of the experiment, we found that the implants were not completely degraded. After extraction of paclitaxel from the tumor, HPLC detection showed that the Taxol group mice had a small amount of paclitaxel residues, whereas the paclitaxel-loaded 30% NBCA and paclitaxel-loaded 40% NBCA groups had approximately 110 μg/mouse paclitaxel residues. In the animal model of liver cancer, residues of 4.5 μg/mouse or 40 μg/mouse epirubicin remained in the epirubicin group and the epirubicin-loaded 50% NBCA group, respectively (Figure 5S). This result is consistent with the experimental result showing NBCA implant biodegradability *in vivo*.

Systematic toxicity was evaluated by measuring body weight changes over time during the treatment period. Significant weight loss was not observed in any of the six groups (Supplemental materials).

### H&E assay

**Figure 6.**
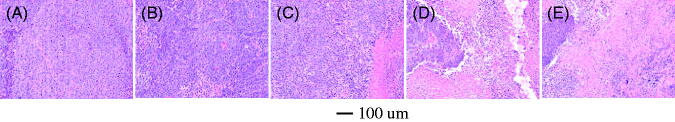
H&E-stained tumor tissues harvested from mice with different treatments. (A) Saline; (B) INEI; (C) taxol; (D) paclitaxel-loaded INEI (30% NBCA); and (E) paclitaxel-loaded INEI (40% NBCA).

H&E staining is shown in Figure 6. A large number of tumor cells were observed in the saline and NBCA-treated groups. The group treated with Taxol showed fewer tumor cells, although the tumors tended to occur later in the experiment. In the paclitaxel-loaded implant groups, only a few living tumor cells could be observed, whereas most of the tumor tissue showed characteristics of necrosis.

## Discussion

The greatest problem associated with available anticancer drugs is their inability to specifically target tumor cells. Thus, only a small part of the drugs will be enriched in the tumor tissue via intravenous injections into the body, and the presence of the drugs released systemically throughout the body can cause serious side effects. As a result, researchers have attempted to improve this situation. The development of antibody drugs is an important direction of anti-tumor drug research, and it has achieved great success. For example, cetuximab and panitumumab target epidermal growth factor receptor (EGFR) to treat colorectal cancer (Braig et al., 2015), trastuzumab targets HER2 to treat breast cancer (Mayor, 2006), and rituximab targets CD20 to treat leucocythemia (Arzoo et al., 2002). Small-molecule targeted anti-tumor drugs have also been greatly advanced (Nishimura et al., 2015). Imitinib is a tyrosine–kinase inhibitor used in the treatment of multiple cancers, most notably Philadelphia chromosome-positive (Ph+) chronic myelogenous leukemia (CML). Similar to erlotinib, Gefitinib is an EGFR inhibitor that inhibits signaling via EGFR in target cells, and is used for treating certain breast, lung, and other cancers. However, the development of new antibody drugs and small-molecule drugs is difficult primarily because tumor cell-specific biomarkers have not been identified and related treatments lack high selectivity (Liu & Kurzrock, 2015). Thus, further progress depends on basic research development.

Currently, hundreds of chemotherapy drugs have been approved for clinical treatment; however, many of these drugs exhibit strong cytotoxicity or induce numerous side effects that may lead patients to abandon treatment. Strategies have been explored to reduce side effects and enhance anti-tumor effects, such as coupling drugs with antibodies, preparing a “biological bomb” with human albumin or PEG, enhancing drug solubility, reducing the removal efficiency of drugs via blood, or preparing drugs into nanoparticles (Maluccio & Covey, 2012) for the use in cancer therapy by intravenous injection. However, the improvement of therapeutic efficiency of the above methods appears to be limited because of the morphological and physiological abnormalities of tumors (Durymanov et al., 2015).

Under the guidance of imaging technology, anti-tumor drugs can be injected directly into tumors, which allow the drugs to form a reservoir and be slowly released, and these treatments may represent an ideal tumor therapy methodology. Commercially important cyanoacrylate monomers include methyl-2-cyanoacrylate, ethyl-2-cyanoacrylate, *n*-butyl-2-cyanoacrylate, and 2-octyl-2-cyanoacrylate. Cyanoacrylates are colorless clear liquids that rapidly polymerize in the presence of water, or more specifically, in the presence of trace amounts of hydroxide ions. Therefore, any slightly alkaline surface with trace amounts of adsorbed water will cure cyanoacrylates by initializing rapid polymerization. Reports indicate that NBCA is a biodegradable, biocompatible, non-toxic material that is widely used as a medical adhesive for wounds (Morton et al., 1988). NBCA is also used for the embolization of cerebral arteriovenous malformations (Duffner et al., 2002) and gastric variceal bleeding (Mosli et al., 2013). In addition, NBCA also has been used to prepare drug-containing nanoparticles used for drug delivery (Tomita et al., 2011).

In this study, we used NBCA as an injectable *in situ*-forming implant triggered by anions contained in body fluids or blood for tumor chemotherapy. Recently, different types of *in situ*-forming gel or implant systems have been reported, and each system has its own advantages and drawbacks. We found that NBCA can be dissolved in ethyl oleate and quickly solidified via polymerization after adding PBS or saline. With increasing NBCA concentrations, the hardness of the solidified material also increased. When the mixture is injected into animal liver tissue, the injection site hardens in a few seconds. This result shows that this type of material can rapidly solidify after contacting with various anions in body fluids (Supplemental materials). After treatment by ultrasound and other methods, the drugs are observed as nanometer or micron-size particles in ethyl oleate, and they do not change size or shape for several months at low temperature. FESEM showed that with increases in the concentration of NBCA, smaller pores were formed after the implant solidified. We believe that these smaller pores can delay drug diffusion into the body fluid when the NBCA and drug/ethyl oleate mixture is injected into the body and solidifies. The drug's *in vitro* release test validated this hypothesis. A considerable amount of the paclitaxel in the dialysis bag had been distributed to the buffer within the first 6 d, whereas less than a quarter of the paclitaxel in the 30% NBCA implant was released. For hydrophilic drugs, NBCA implants also showed an excellent sustained-release effect *in vitro*. In addition, NBCA greatly reduced the burst release of the hydrophilic drug, with approximately 10% of the total amount of the drug released in the first day in the NBCA implant group versus approximately 40% of the total drug released in the control group.

The biodegradation of an implant influences the mode and rate of drug release *in vivo* as well as its clinical applications. Non-degradable implants are not used for deep injections into the human body because the implant must be removed via surgery after complete drug release, which results in damage to the patient. We studied the degradation of the NBCA implant *in vivo* and found that the degradation time of the NBCA implant was prolonged with an increase in the NBCA concentration. The time for complete degradation of the 50% NBCA implant was 8 weeks *in vivo*. Depending on the tumor size, the proportion of NBCA in the implant can be adjusted to generate implants with different degradation times, and the ability to alter the degradation times of implants will be an important advantage for clinical applications.

To observe the sustained-release effect of small-molecule drugs from NBCA, NBCA implants containing the fluorescent dye DiR were injected into breast cancer xenograft tumors, and the fluorescence intensity in the mice was observed via *in vivo* imaging. The results showed that the mice injected with implants exhibited strong fluorescence that was sustained for 20 d. In addition, 3 h after injection, the control group showed similar fluorescence intensities in the tumor and in other parts of the body. This result shows that the NBCA implants can indeed slow the release of small molecules to the tumors. Anti-tumor experiments showed that the growth inhibition rate of breast tumors from the paclitaxel-loaded 40% NBCA implant was 80%, and certain mice tumors were completely ablated. For the epirubicin-loaded 50% NBCA implant, the growth inhibition rate of liver tumors was 58%.

In conclusion, we developed a novel injectable *in situ*-forming implant system that can be triggered to solidify by body fluid and blood and shows an excellent sustained-release effect of hydrophobic and hydrophilic drugs. The formulation and implementation of this system is simple, and it holds great promise for efficient intratumoral chemotherapy in clinical applications.

## Supplementary Material

Supplementary_Material.docx

## Notes

**Supplementary material available online**
