# Supplementary material for: N-Butyl-2-cyanoacrylate-based injectable and in situ-forming implants for efficient intratumoral chemotherapy
Source: Drug Deliv. 2017 Apr 25;24(1):729–36. doi: 10.1080/10717544.2017.1309478 (PMC8240990; doi:10.1080/10717544.2017.1309478)
Supplement: Supplementary_Material.docx [file IDRD_A_1309478_SM1649.docx]

## Result


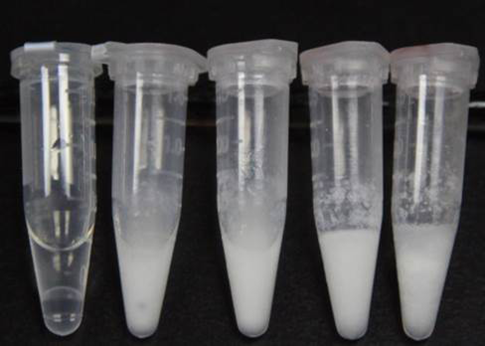


E

D

C

B

A

Fig.S1. Implants containing different concentration of NBCA. A: ethyl oleate. B: paclitaxel-10% NBCA of INEI. C: paclitaxel-20% NBCA. D: paclitaxel-30% NBCA. E: paclitaxel-40% NBCA.

400 μL of different concentration of NBCA were added into each 1.5 ml Eppendorf tube, 10 μL of saline were then added and the tube was vortexed for 10 s separately. With the increase of NBCA concentration, the hardness of the solidified material is also increased. When the concentration of NBCA is below 20%, the material is hardly solidified.


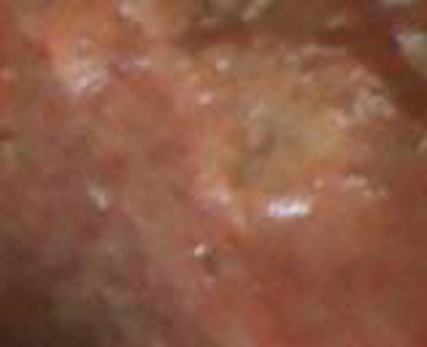


A


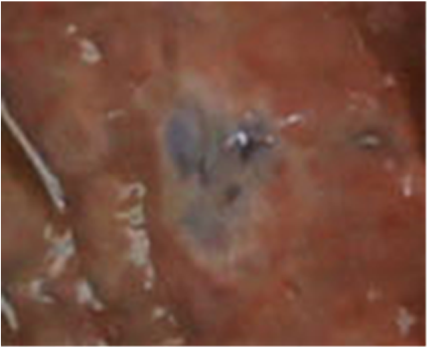


B

Fig. S2. Characteristic of paclitaxel-NBCA gels in fresh liver. A: paclitaxel-NBCA. B: paclitaxel-NBCA with methylene blue.

100 μL paclitaxel-30% NBCA and 100 μL blue paclitaxel-30% NBCA (with 0.1mg coomassie brilliant blue) were separately injected into two pieces of fresh pig livers. Both the two kinds of NBCA solidified quickly after injection.

Fig. S3. Time course of changes of tumor volumes upon INEI treatment. Mice were subjected to A: a single intratumoral injection of 30 μL of saline, 30% NBCA of INEI, Taxol (containing 0.3 mg paclitaxel), 0.3 mg paclitaxel-30% NBCA of INEI, 0.3 mg paclitaxel-40% NBCA of INEI, or B: a single intratumoral injection of 30 μL of saline, 30% NBCA of INEI, epirubicin solution (containing 0.15 mg epirubicin), 0.15 mg epirubicin-50% NBCA of INEI. Volumes of tumors were measured with a caliper every two days. The volume of tumor was calculated by the formula: V= (length) × (width) 2 /2, (L (mm) and W (mm) are the tumor dimensions at the longest point and widest point). After 20 days, all the mice were sacrificed, and the tumors from each group were weighed, **p<0.05, ***p<0.01.


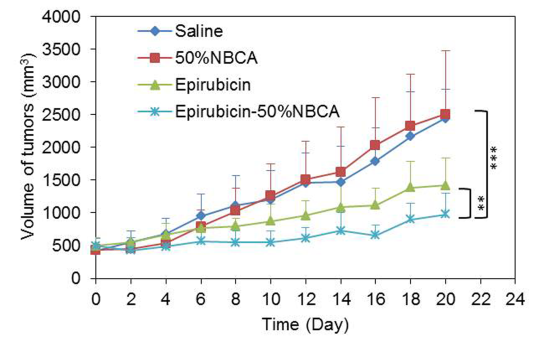


B


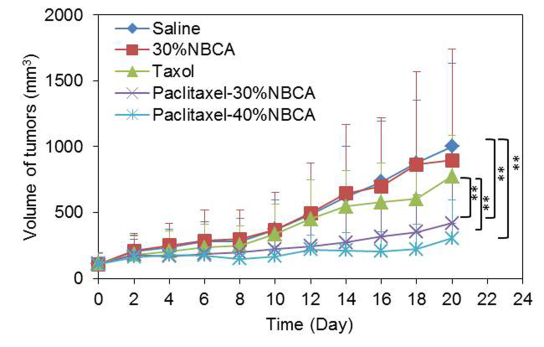


A

Fig. S4. Time course of tumor masses upon INEI treatment. Mice were subjected to A: a single intratumoral injection of 30 μL of saline, 30% NBCA of INEI, Taxol (containing 0.3 mg paclitaxel), 0.3 mg paclitaxel-30% NBCA of INEI, 0.3 mg paclitaxel-40% NBCA of INEI, or B: a single intratumoral injection of 30 μL of saline, 30% NBCA of INEI, epirubicin solution (containing 0.15 mg epirubicin), 0.15 mg epirubicin-50% NBCA of INEI, **p<0.05, ***p<0.01.


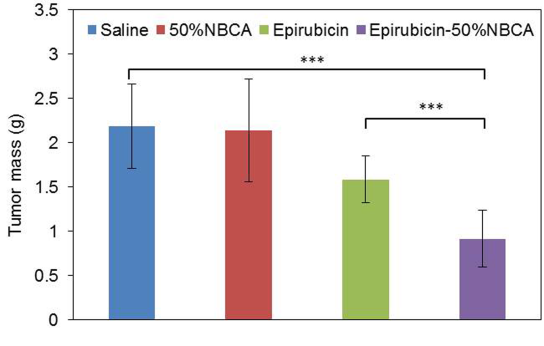


B


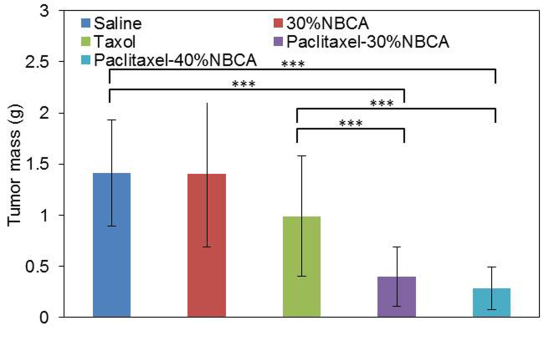


A

Fig. S5. Drug residues in animal tumors. A: Paclitaxel-loaded INEI for human xenograft breast tumor therapy. B: Epirubicin-loaded INEI for human xenograft liver tumor therapy. Each data point represents the mean± S.D. of triplicate measurements (n = 3).


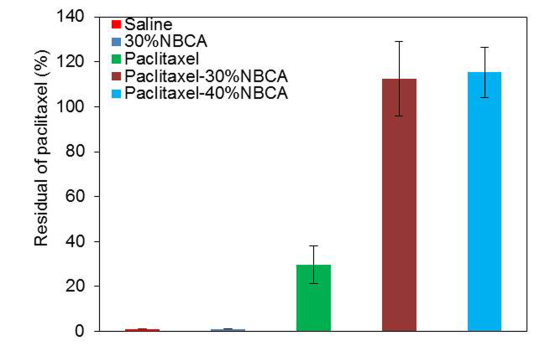


A


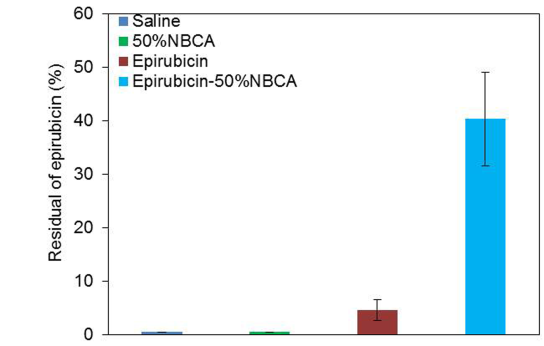


B


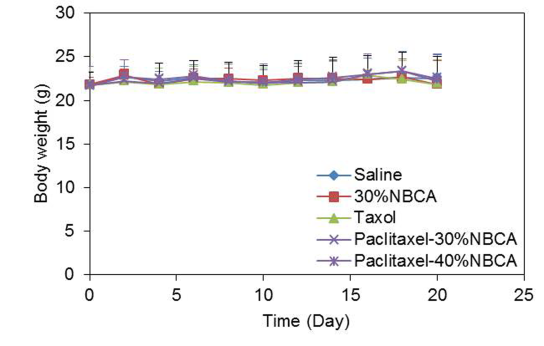


A


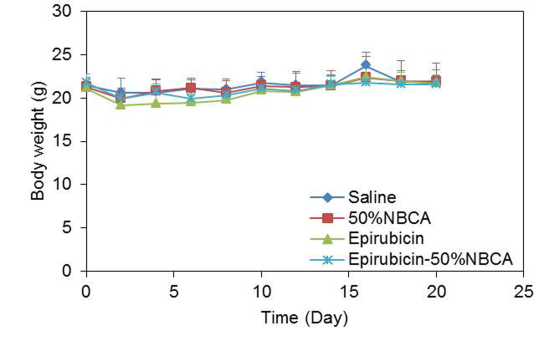


B

Fig. S6. Time course of changes of body weights. Mice were subjected to A: a single intratumoral injection of 30 μL of saline, 30% NBCA of INEI, Taxol (containing 0.3 mg paclitaxel), 0.3 mg paclitaxel-30% NBCA of INEI, 0.3 mg paclitaxel-40% NBCA of INEI, or B: a single intratumoral injection of 30 μL of saline, 30% NBCA of INEI, epirubicin solution (containing 0.15 mg epirubicin), 0.15 mg epirubicin-50% NBCA of INEI. Before treatment, the body weight of each mouse was measured, and after drug administration the body weight of each mouse was measured every two days. The physiological state of mice was observed during the treatment process. The data of the weight was recorded as a function of time.

Saline

INEI

Taxol

Palitaxel-30% NBCA (three injections)

Palitaxel-30% NBCA (two injections)

Palitaxel-30% NBCA (one injection)


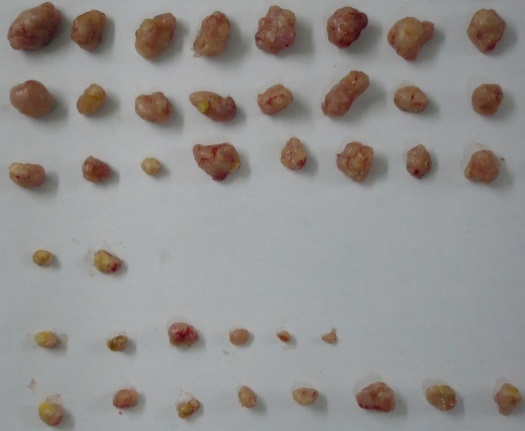


Fig. S7. Tumors of different groups. A-C: after a single intratumoral injection of 30 μL of saline, 30% NBCA of INEI, Taxol (containing 0.3 mg paclitaxel), D: three 0.3 mg paclitaxel-30% NBCA of INEI injections at each of the first three weeks separately, E: two 0.3 mg paclitaxel-30% NBCA of INEI injections at each of the first two weeks separately, F: one 0.3 mg paclitaxel-30% NBCA of INEI injection at the first week.
